# Mass Spectrometric Characterization of Histone H3 Isolated from *in-Vitro* Reconstituted and Acetylated Nucleosome Core Particle

**DOI:** 10.5702/massspectrometry.A0090

**Published:** 2020-10-31

**Authors:** Kazumi Saikusa, Haruna Hidaka, Shunsuke Izumi, Satoko Akashi

**Affiliations:** 1 National Metrology Institute of Japan (NMIJ), National Institute of Advanced Industrial Science and Technology (AIST), 1–1–1 Umezono, Tsukuba, Ibaraki 305–8563, Japan; 2 Graduate School of Science, Hiroshima University, 1–3–1 Kagamiyama, Higashi-Hiroshima, Hiroshima 739–8526, Japan; 3 Graduate School of Medical Life Science, Yokohama City University, 1–7–29 Suehiro-cho, Tsurumi-ku, Yokohama 230–0045, Japan; 4 Graduate School of Integrated Sciences for Life, Hiroshima University, 1–3–1 Kagamiyama, Higashi-Hiroshima, Hiroshima 739–8526, Japan

**Keywords:** nucleosome core particle, histone H3, *in vitro* modification, aminopeptidase digestion, MALDI-ISD experiment

## Abstract

Post-translational modifications (PTMs) of histone N-terminal tails in nucleosome core particle (NCP), such as acetylation, play crucial roles in regulating gene expression. To unveil the regulation mechanism, atomic-level structural analysis of *in-vitro* modified NCP is effective with verifying the PTMs of histones. So far, identification of PTMs of NCP originating from living cells has mainly been performed using mass spectrometry (MS) techniques, such as bottom-up approach. The bottom-up approach is the most established method for protein characterization, but it does not always provide sufficient information on the acetylated sites of lysine residues in the histone tails if trypsin digestion is carried out. For histone proteins, which have many basic amino acids, trypsin generates too many short fragments that cannot be perfectly analyzed by tandem MS. In this study, we investigated the *in vitro* acetylation sites in the histone H3 tail using a top-down sequence analysis, matrix-assisted laser desorption/ionization in-source decay (MALDI-ISD) experiment, in combination with aminopeptidase digestion. Aminopeptidase can cleave peptide bonds one-by-one from the N-terminus of peptides or proteins, generating N-terminally truncated peptides and/or proteins. As a result, it was identified that this method enables sequence characterization of the entire region of the H3 tail. Also, application of this method to H3 in *in-vitro* acetylated NCP enabled assigning acetylation sites of H3. Thus, this method was found to be effective for obtaining information on *in-vitro* acetylation of NCP for structural biology study.

## INTRODUCTION

In the eukaryotic nucleus, DNA is organized into packed chromatin, whose basic structural unit is nucleosome core particle (NCP).^1–3)^ The NCP is composed of an octamer of the four histones (H2A, H2B, H3, and H4) wrapped around *ca.* 147 base pairs (bp) of DNA. The N-terminal region of each histone, histone tail, contains high proportion of basic amino acids, and is unstructured. It has been demonstrated that post-translational modifications (PTMs) of the histone tails play crucial roles in regulating gene expression.^4–6)^ Histone acetylation, one of the PTMs of NCP, triggers translational activation.^7)^ When the basic amino acids, such as lysine (Lys), in histone tails are acetylated, it is expected that electrostatic interactions between the basic histones and the negatively charged DNA are reduced, leading to loosening the NCP structure. There are various histone acetyltransferases (HATs), which catalyze the transfer of an acetyl moiety from acetyl coenzyme A (Acetyl-CoA) onto the ε-amino group of Lys; they regulate nucleosome assembly and the folding of chromatin by working at an appropriate situation.^7)^ Also, multiple modifications work cooperatively and simultaneously for these regulations. Therefore, in order to correctly understand the functional changes brought about by each modification, it is necessary to reproduce each modification *in vitro* and characterize the function and structure relationship with controlling the extent of modification of NCP. To determine the atomic-level structure of modified NCP, X-ray crystallography has mainly been used, while mass spectrometry (MS) is an essential tool for identifying these PTMs.^8,9)^

There are three MS-based approaches for analyzing protein sequence: bottom-up, top-down, and middle-down.^8,9)^ In the bottom-up approach, proteins are digested into peptides with trypsin, a protease mainly used, and the peptides are analyzed by MS and tandem MS (MS/MS). Using the information obtained by MS and MS/MS analyses, the sequence of each peptide is characterized. In the top-down approach, proteins are isolated and fragmented within the mass spectrometer to obtain sequence information, which can be analyzed by database searching. In contrast, the middle-down approach is the intermediate between the bottom-up and the top-down approaches; proteins are digested into peptides with a protease such as Glu-C, which usually generates polypeptides consisting of 30–40 amino acids. The most popular and established approach is the bottom-up approach. However, the lengths of peptides obtained by tryptic digestion are often too short to analyze by MS/MS, since histone tails are rich in Lys and arginine (Arg). In addition, it is difficult to completely characterize the global status of the PTM patterns of a particular protein, because the relationship between multiple modifications cannot be identified from the sequence analysis of many short peptides if they contain just a single PTM. On the other hand, the other two approaches can reveal the relationship of co-existing modifications, because relatively large peptides/proteins can be analyzed.^10–12)^ To cleave large polypeptides and proteins, collision-induced dissociation (CID), the major fragmentation method equipped with most of the tandem mass spectrometers, is not appropriate and cannot provide enough sequence information for them when used in combination with these two approaches. For the top-down and the middle-down approaches, other fragmentation methods that cleave peptide bonds by radical, such as electron capture dissociation (ECD)^13)^ or electron transfer dissociation (ETD),^14)^ are effective.

In the ECD and the ETD mechanism, electron attachment and transfer occur at positively charged sites, such as basic amino acids, and the π* antibonding orbital of peptide bond, producing fragment ions around these sites.^15)^ However, it is not easy to identify all fragment ions derived from a large protein. For sequence analysis of large peptides/proteins, another alternative is matrix-assisted laser desorption/ionization in-source decay (MALDI-ISD). To generate informative fragment ions for sequence analysis, the choice of matrix for MALDI-ISD experiment is very important.^16)^ Regardless of matrix selection, the fragment ions observed in the low *m*/*z* region mainly provide sequence information from the N- or C-terminus. Therefore, the MALDI-ISD experiment is considered to be a promising tool for characterizing histone PTMs, as reported by Kwak and Dohmae.^17)^

Here, we characterized the sequence of H3, which has the longest histone tail among four histones, with MALDI-ISD experiment combined with aminopeptidase digestion. Aminopeptidase can cleave peptide bonds from the N-terminus of peptides or proteins, resulting in N-terminally truncated peptides and/or proteins. By this method, we could successfully obtain the sequence information of a long tail region of H3. Furthermore, this method was applied to H3 in NCP with/without acetylation by HAT p300 *in vitro*. It enabled complete sequence characterization of the acetylated H3 tail region in reconstituted NCP *in vitro*, which would help to facilitate structural biology of epigenetic regulation.

## EXPERIMENTAL

### Preparation of acetylated NCP

Histone octamer consisting of each two molecules of histone H2A, H2B, H3 and H4 was prepared by refolding recombinant histone monomers followed by purification *via* gel filtration using a Superdex200 (GE Healthcare, UK Ltd., Buckinghamshire, UK), as described in previous reports.^18–20)^ NCP was reconstituted with a histone octamer and Widom 601 DNA (147 bp)^21)^ by salt dialysis method.^18–20)^

Acetylation of NCP was performed using HAT p300 (Enzo Life Sciences, Farm-ingdale, NY) at a 20 : 1 substrate:enzyme ratio (w/w). The mixture of the NCP and HAT p300 was incubated in 50 mM Tris–HCl (pH 8.0), 10% glycerol, 0.1 mM EDTA, and 1 mM dithiothreitol (DTT) in the presence of 1 mM Acetyl-CoA at 4°C for 18 h. The extent of acetylation of each histone was determined by MALDI-TOF MS.

### Separation of histones from NCP by HPLC

For histone isolation from NCP, denaturing buffer (1.6 mM NaCl, 2.1 M guanidine HCl, and 0.5% trifluoroacetic acid (TFA)) was first added to NCP. Denatured NCP was then applied to a C18 high performance liquid chromatography (HPLC) trap column (2.1×10 mm, Waters, Milford, MA) at a flow rate of 0.4 mL/min. Each histone was separated with a C8 reverse phase (RP) column (2.1×150 mm, 5 μm particle size; GL sciences, Japan), which was sequentially connected to the C18 trap column, at room temperature. A 100-μL sample was injected and protein peaks were detected at a UV wavelength of 215 nm. The mobile phases A (5% acetonitrile with 0.1% TFA) and B (100% acetonitrile with 0.1% TFA) were delivered at a flow rate of 0.4 mL/min using the following gradient program: 0 to 5 min, 0% solvent B; 5 to 10 min, 0–37% solvent B; 10 to 15 min, 37% solvent B; 15 to 30 min, 37–42.30% solvent B; 30 to 40 min, 42.30–49.50% solvent B; 40 to 50 min, 49.50–90% solvent B; 50 to 60 min, 90% solvent B; 60 to 65 min, 90–0% solvent B. The fraction including each histone was collected manually. Each fraction was concentrated under vacuum before digestion or MALDI-ISD experiments. The fractions were UV-measured by nanodrop 1000 (Thermo Fisher Scientific, Boston, MA), and their concentration was calculated. In the case of digestion, the solvent of each fraction was exchanged to MilliQ using ultrafiltration.

### Digestion of H3 with Aminopeptidase

The protein was digested with *Aeromonas proteolytica* aminopeptidase (MEROPS M28.002, Sigma-Aldrich, St. Louis, MO).^22–25)^ A 1 μL of the protease solution in 10 mM Tris–HCl (pH 8.0) (0.2 U/μL) was added to 1 nmol of H3 in 10 mM Tris–HCl and the sample solution was kept standing at 25°C to obtain N-terminally truncated H3. Digestion products were analyzed with MALDI-TOF MS.

### LC-MS

For bottom-up approach, LC-MS measurement was performed using LTQ Orbitrap XL (Thermo Fisher Scientific, Boston, MA) mass spectrometer equipped with a nano-LC in positive-ion mode. A 5 μL of H3 solution (100 μM, 500 pmol) in 50 mM ammonium bicarbonate was added to a 1 μL of trypsin solution (0.1 mg/mL) and kept at 37°C for overnight. After the digestion, the tryptic peptides were desalted and concentrated with Zip Tip C18 (Merck, Germany), and then evaporated. The dried tryptic peptides were added to a 10 μL of 0.1% TFA. The solution of tryptic peptides was applied to a C18 trap column (300 μm I.D.×5 mm, Thermo Fisher Scientific, Boston, MA) at a flow rate of 200 nL/min. Each tryptic peptide was separated with a C18 RP column (75 μm I.D.×120 mm, 3 μm particle size; Nikkyo Technos, Co., Ltd., Japan), which was sequentially connected to the C18 trap column, at room temperature. The mobile phases A (0.1% formic acid (FA)) and B (100% acetonitrile with 0.1% FA) were delivered at a flow rate of 200 nL/min using the following gradient program: 0 to 3 min, 4% solvent B; 3 to 35 min, 4–35% solvent B; 35 to 36 min, 35–90% solvent B; 36 to 45 min, 90% solvent B; 45 to 45.01 min, 90–4% solvent B; 45.01 to 60 min, 4% solvent B. The range of *m*/*z* in each measurement was 300–1500. The data was processed using Proteome Discoverer 1.4 (Thermo Fisher Scientific, Boston, MA) by MASCOT software. MASCOT was set up to assign the fragment ions to the human histone sequences we used. The maximum number of missed cleavage sites was set to 2.

### MALDI-MS

For MALDI-ISD experiment, 1,5-diaminonaphtalene (DAN) (10 mg/mL) was dissolved in water/acetonitrile (1 : 1, v/v) with 0.1% TFA. A 1 μL of sample solution (*ca.* 30–40 pmol) was deposited onto a stainless-steel plate and the solvents were evaporated. Then, a 1 μL of matrix solution was deposited onto the stainless-steel plate and the solvents were evaporated. MALDI-ISD measurement was performed using AXIMA CFR (Shimadzu/Kratos, U.K.) or Biflex IV (Bruker, Germany) mass spectrometers equipped with a nitrogen UV laser (337 nm) in linear positive-ion mode. The laser power was optimized in order to obtain MALDI-ISD spectra with high signal-to-noise ratios (*S*/*N*) for the ISD ions.

For MALDI-TOF experiment, sinapinic acid (SA) (10 mg/mL) was dissolved in water/acetonitrile (1 : 1, v/v) with 0.1% TFA. A 1 μL of sample solution (*ca.* 30–40 pmol) was mixed with a 1 μL of matrix solution and then deposited onto a stainless-steel plate and the solvents were evaporated. MALDI-TOF mass spectra were recorded using the same instruments as MALDI-ISD experiments. The spectra were calibrated with peptides (angiotensin II (*m*/*z* 1046.5418), angiotensin I (*m*/*z* 1296.6848), substance P (*m*/*z* 1347.7354), bombesin (*m*/*z* 1619.8223), adrenocorticotropic hormone (ACTH) clip 1–17 (*m*/*z* 2093.0862), and ACTH clip 18–39 (*m*/*z* 2465.1983)) or proteins (insulin (*m*/*z* 5734.51), ubiquitin I (*m*/*z* 8565.76), cytochrome *C* (*m*/*z* 12360.97(1+), 6180.99 (2+)), and myoglobin (*m*/*z* 16952.30 (1+), 8476.65 (2+))) (Bruker, Germany).

## RESULTS AND DISCUSSION

### Sequencing H3 tail using bottom-up and top-down approaches

**Figure 1:**
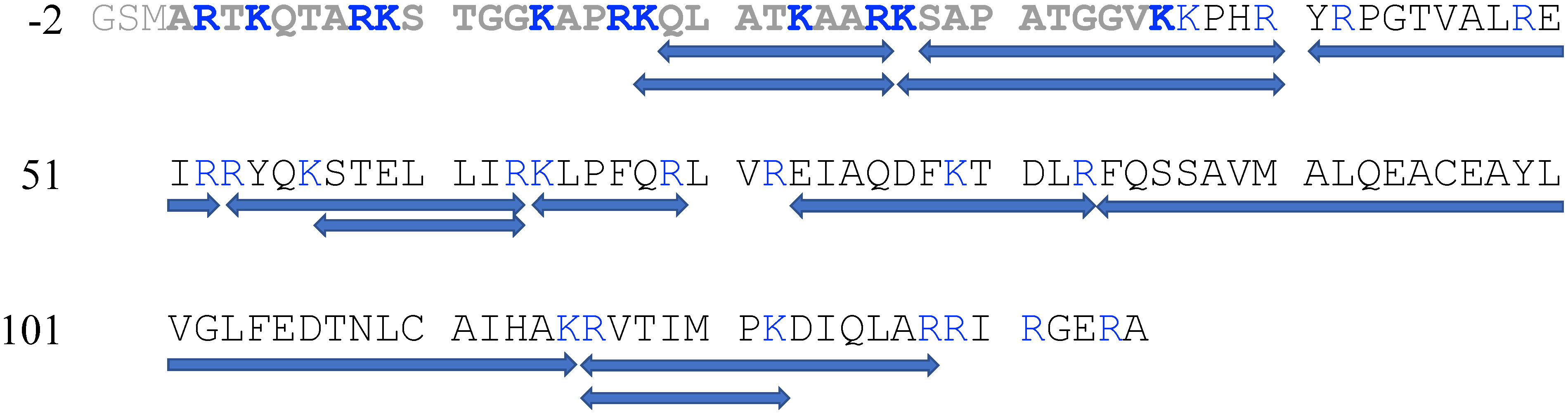
Fig. 1. Peptide map of H3 generated by tryptic digestion.

**Figure 2:**
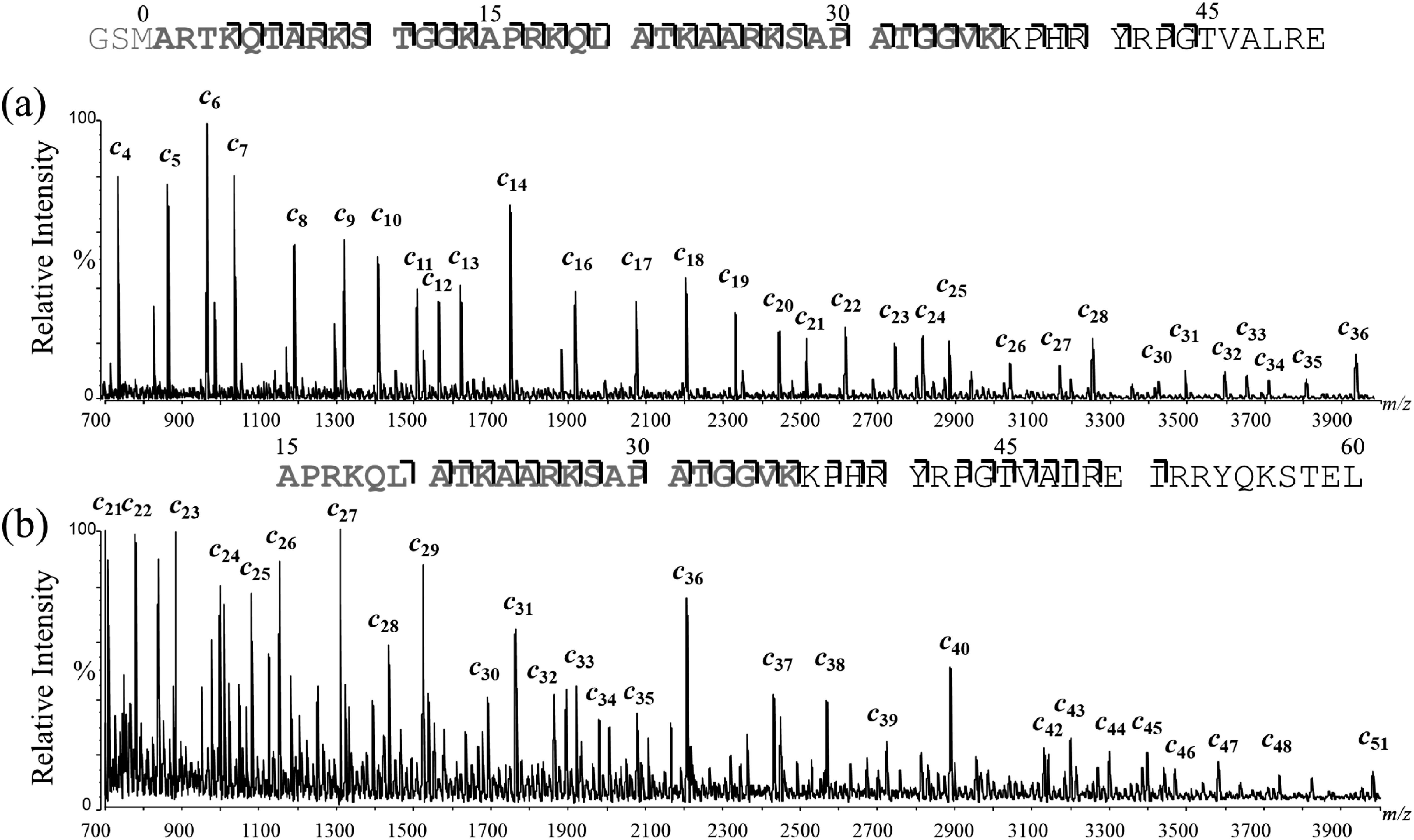
Fig. 2. MALDI-ISD mass spectra of (a) recombinant H3 and (b) aminopeptidase-digested H3.

Figure 1 shows the amino acid sequence of human histone H3 used in the present study. Gray characters (36 residues from alanine1 (Ala1)) in Fig. 1 indicate the tail region suggested by the X-ray crystallography analysis of reconstituted NCP because this region was invisible in the structure.^26)^ Here we defined this region as the histone H3 tail. In the present study, recombinant H3 with a histidine tag was expressed in *Escherichia coli*. After removal of the histidine tag by proteolysis with thrombin, purified H3 retained the linker sequence of GSM, which connected the histidine tag and the H3 sequence, at the N-terminus of the protein. First, the sequence of H3 was analyzed by the bottom-up approach. As indicated in Fig. 1, the sequence information could be obtained for the region of 18–128th residues except for 70th–72nd residues. Although acetylation or methylation of Lys4, Lys9, and Lys14 is responsible for regulation of transcription initiation, the sequence information for 20 amino acids from the N-terminus could not be obtained.^6)^ To obtain the sequence information of this region, we first examined MALDI-ISD of recombinant H3, as shown in Figs. 2a and S-1. DAN, a reducing matrix, was used for this experiment to observe *c* or *z*+2 ions similar to ECD or ETD.^27–29)^ Observed peaks were assigned to *c* ions, corresponding to the fragments from the N-terminus to glycine44 (Gly44) of H3, demonstrating that the complete sequence information for the H3 tail region can be obtained by the MALDI-ISD experiments.

Gray bold characters indicate residues that could not be modeled in the crystal structure of the human NCP (PDB I.D. 3AFA).^26)^ Blue characters indicate the basic amino acids in H3. Blue arrows indicate the tryptic peptides obtained by the bottom-up approach. The linker sequence of “GSM” is also indicated at the N-terminus of the H3 sequence.

The mass spectra were obtained by AXIMA-CFR. Gray bold characters indicate residues that could not be modeled in the crystal structure of the human NCP (PDB I.D. 3AFA).^26)^ The numbers used for annotation of *c* ions start from Ala1 in the original H3 sequence shown in Fig. 1. Figure 2a shows the expanded spectrum for *m*/*z* 700–4000 while Fig. S-1 shows the same spectrum in the range of *m*/*z* 700–5000.

Considering that H3 exists as a component of NCP and undergoes various PTMs in living cells, it is necessary to establish a method that enables sequence characterization of H3 isolated from NCP. Consequently, H3 was isolated from the reconstituted NCP by HPLC and then similarly analyzed by MALDI-ISD. About 160 pmol of each histone was recovered from 100 pmol of the injected NCP by the HPLC system. The recovery yield was estimated to be approximately 80% because two molecules of each histone protein were contained in each NCP. Figure S-2 shows MALDI-ISD mass spectrum of isolated H3 by HPLC. The observed peaks were identified as *c*_4_–*c*_17_ ions. Histone H3 is highly basic due to many Arg and Lys residues especially in the tail region. Within NCP, H3 makes direct contacts with negatively charged DNA. To dissociate histones from DNA, NaCl and an unfolding reagent, such as guanidine HCl, were added to the NCP sample and then desalted using the trap column. However, even desalted with the trap column, basic residues in histones would have retained chloride ions due to their high basicity. ISD requires a positive charge on the analyte protein to start the reaction. When basic amino acids form salts with chloride ions, it is difficult to obtain sequence information of the protein by ISD due to loss of basicity. The *S*/*N* of the fragment ions are reduced for the protein associated with salts. Thus, isolated H3 from the reconstituted NCP presented poor *S*/*N* of the fragment signals in ISD spectrum. Therefore, it would have been difficult to analyze the whole tail regions of H3 in NCP only by MALDI-ISD.

### Sequencing H3 tail using MALDI-ISD in combination with aminopeptidase digestion

To obtain the sequence information of H3 tails in NCP in detail, we applied MALDI-ISD in combination with aminopeptidase digestion. Aminopeptidase cleaves peptide bonds one-by-one from the N-terminus of peptides or proteins, resulting in N-terminally truncated peptides and/or proteins. In the case of histone H3, it is expected that the sequence information of the region that cannot be read out with a simple experiment only by MALDI-ISD can be obtained by the combination of aminopeptidase digestion with MALDI-ISD-MS. Figure S-3 shows MALDI mass spectra of aminopeptidase digests of H3. The observed *m*/*z* value of 13808 was *ca.* 1730 smaller than the original *m*/*z* value in Fig. S-3a. It was also found that aminopeptidase digestion for 6 h was enough to remove the N-terminal 17 residues from the histone H3 protein, as shown in Fig. S-3c. The decreased mass value was consistent with the characteristics of aminopeptidase, which does not cleave the peptide bond connecting to the proline imino group. The N-terminal amino acid of the newly generated polypeptide is Ala15 of the H3 sequence (Fig. 1). Consequently, we subjected this N-terminally truncated H3 to the MALDI-ISD experiment. As shown in Fig. 2b, it was possible to obtain the sequence information from Ala21 to isoleucine51 (Ile51) residues of H3, which could not be analyzed from the ISD mass spectrum of intact H3 prepared form reconstituted NCP. In Fig. 2b, the baseline of the ISD spectrum was a little noisy. This might be due to the fragmentation of the peptides originated from non-specific cleavage of aminopeptidase.

### Identification of H3 in acetylated NCP

**Figure 3:**
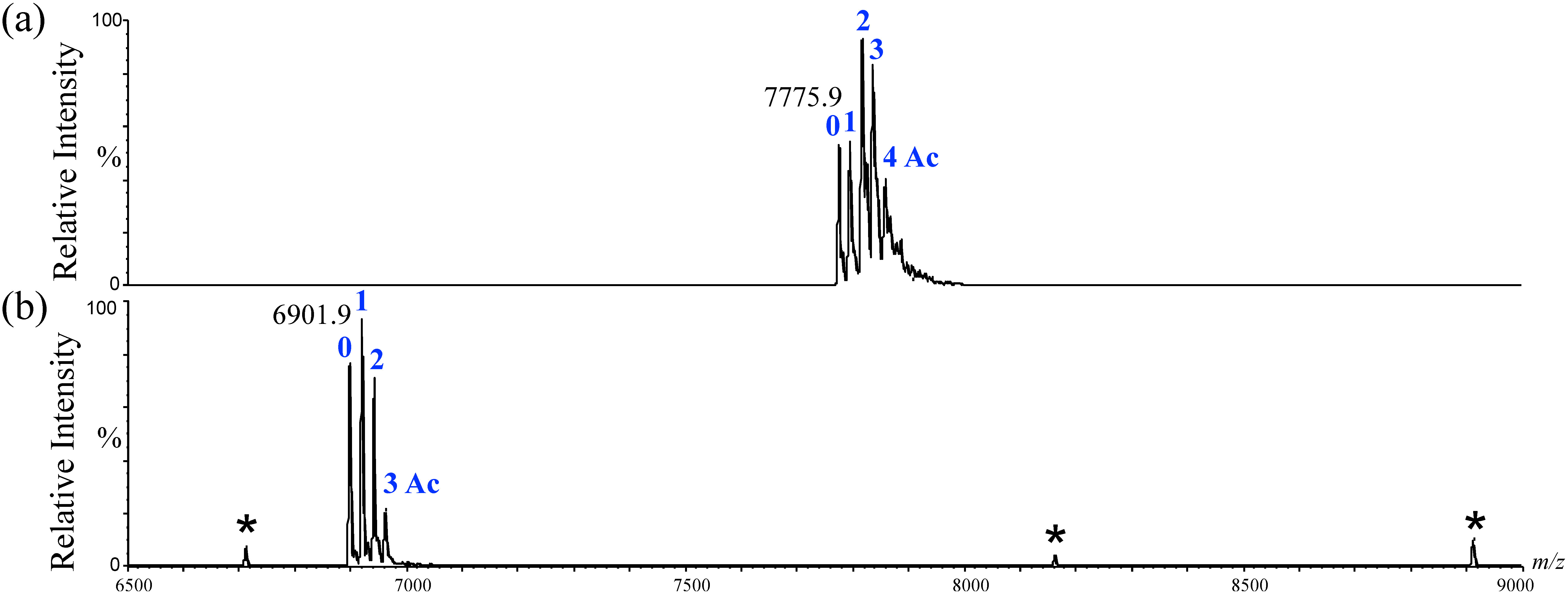
Fig. 3. MALDI mass spectra of acetylated H3 (a) before and (b) after aminopeptidase digestion.

**Figure 4:**
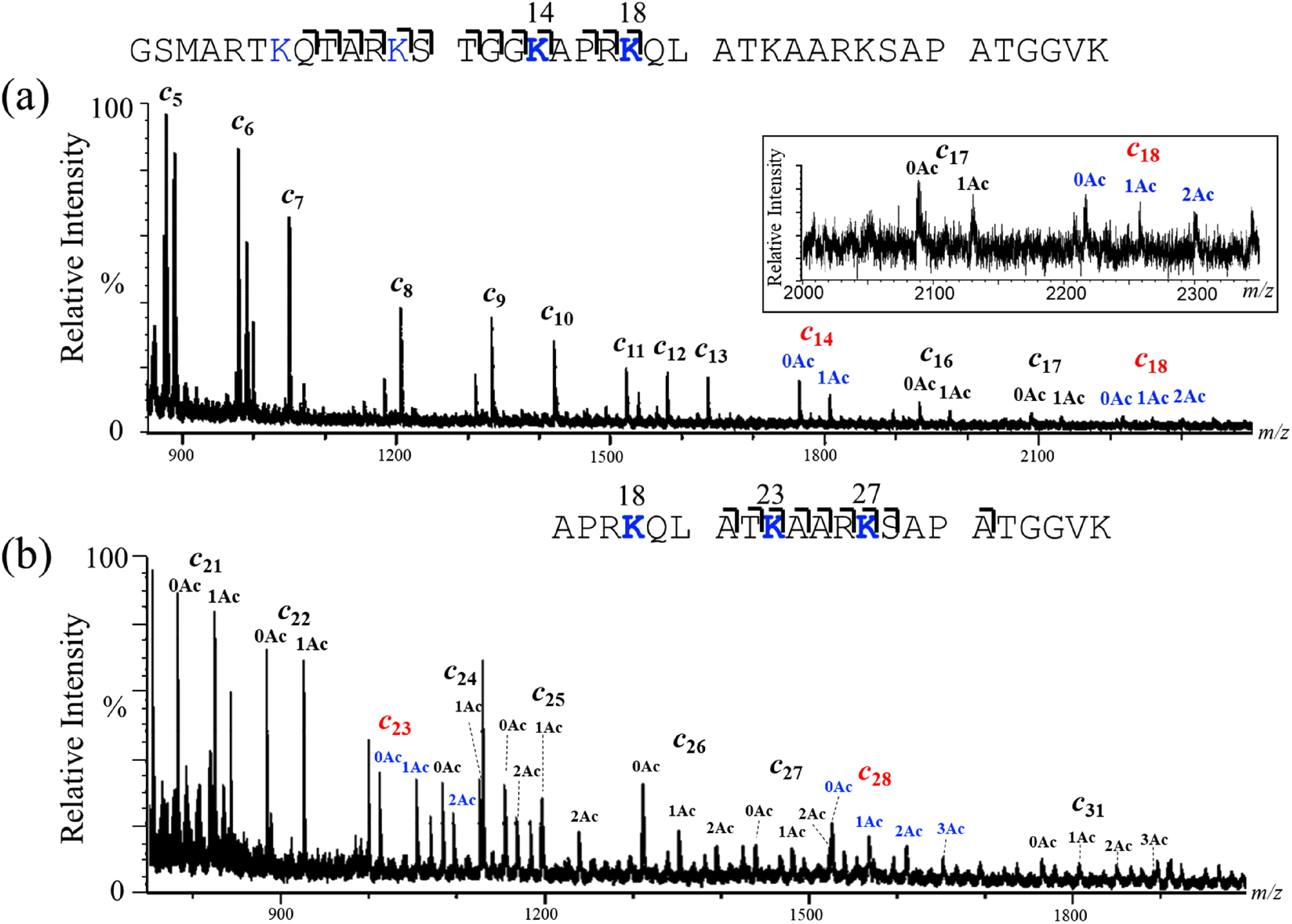
Fig. 4. MALDI-ISD mass spectra of acetylated H3 (a) before and (b) after aminopeptidase digestion.

Next, in order to examine whether the modified sites of H3 can be identified by the above method, we performed acetylation of histone proteins in reconstituted NCP, and then investigated the acetylated sites in H3. Figure 3 shows MALDI mass spectra of acetylated H3 in NCP with/without aminopeptidase digestion. In the mass spectrum of H3 in Fig. 3a, doubly charged peaks observed around *m*/*z* 7700 suggest that H3 was from zero to tetra acetylated. For the N-terminally truncated H3, peaks around *m*/*z* 6900 were assigned to from zero- to tri-acetylated H3, as shown in Fig. 3b. In addition, the acetylation level in the N-terminally truncated H3 was one less, suggesting that there is mono-acetylated Lys from N-terminus to Lys14. Next, MALDI-ISD measurement was performed on these acetylated H3 with/without aminopeptidase digestion, as shown in Fig. 4. In mass spectrum of acetylated H3, as shown in Fig. 4a, observed peaks were identified as *c*_5_–*c*_18_ ions. Among observed *c* ions, *c*_14_–*c*_17_ ions were zero- and mono-acetylated. Also, *c*_18_ ion was zero, mono and di-acetylated, as shown in Fig. 4a inset. Consequently, it was estimated that Lys14 and Lys18 were partly acetylated, while Lys4 and Lys9 were not modified at all. In the mass spectrum of N-terminally truncated H3 (Fig. 4b), observed peaks were assigned to *c*_21_–*c*_31_ ions, showing partly acetylation at Lys18, Lys23 and Lys27. Schiltz reported using microsequence analysis that p300 acetylates Lys14, Lys18 and Lys23 in H3 *in vitro*.^30)^ Also, the extent of acetylation of Lys23 was lower than that of Lys14 and Lys18.^30)^ The difference of acetylation sites and levels between the previous and our studies might have been caused by the difference in the interaction sites of histone and DNA within reconstituted NCP. In the present study, unmodified NCP was first prepared and then it was acetylated with HAT p300. If DNA would not have tightly and uniformly wrapped around the histone octamer in the reconstituted NCP, the positive charges of basic amino acids in histones might interact with negative charges at different positions of DNA strand, resulting in the different acetylation sites and levels of H3.

The mass spectra were obtained by AXIMA-CFR. The peaks with asterisks indicate peptides derived from non-specific cleavage of aminopeptidase. The numbers of acetyl group within the proteins are indicated in blue. Observed *m*/*z* values for the proteins with no acetyl groups are indicated above the corresponding peaks.

The mass spectra were obtained by Biflex IV. The inset panel in Fig. 4a shows expanded mass spectrum in the range of *m*/*z* 2000–2350. The sequence above each mass spectrum shows the fragmentation sites of H3. Blue characters indicate Lys residues in each sequence. The numbers above these sequences indicate the positions of partly acetylated Lys residues. Annotations in blue show the number of acetyl group included in each *c* ion shown in red.

As mentioned above, it was demonstrated that the method combining aminopeptidase digestion and MALDI-ISD is useful for identifying the modification sites of the long tail region of H3. Furthermore, our results demonstrate the importance of verification of the modified sites for each experiment, even the site specificity has already been well characterized. Thus, this method should be effective for characterization of a variety of *in-vitro* modified NCP prepared for structural biology study.

## CONCLUSION

In this study, we characterized the sequence of H3 with MALDI-ISD experiment combined with aminopeptidase digestion. Although H3 is the longest histone with the longest tail region among four histone proteins that consist NCP, it was possible to analyze the entire tail region of H3. We also applied this method to H3 originated from acetylated NCP, and were able to identify the *in-vitro* acetylated sites of H3. Considering that there are many histone variants, such as CENP-A, and important PTMs in H3 related to function of chromatin, this method should be effective to characterize the relationship between function and PTMs of the tail regions of histone H3. Furthermore, we believe that this method would help understanding the transcription activation mechanism by various modifications, which result in structural changes in NCP.

## Abbreviations

NCP, nucleosome core particle; bp, base pairs; PTM, post-translational modification; Lys, lysine; HATs, histone acetyltransferases; Acetyl-CoA, acetyl coenzyme A; MS, mass spectrometry; CID, collision-induced dissociation; Arg, arginine; MS/MS, tandem mass spectrometry; ECD, electron capture dissociation; ETD, electron transfer dissociation; ESI, electrospray ionization; MALDI, matrix-assisted laser desorption/ionization; ISD, in-source decay; DTT, dithiothreitol; TFA, trifluoroacetic acid; HPLC, high performance liquid chromatography; RP, reverse phase; FA, formic acid; DAN, 1,5-diaminonaphthalene; *S*/*N*, signal-to-noise ratio; SA, sinapinic acid; ACTH, adrenocorticotropic hormone; Ala, alanine; Gly, glycine; Ile, isoleucine

## Notes

Mass Spectrom (Tokyo) 2020; 9(1): A0090
