# Supplementary material for: Mass Spectrometric Characterization of Histone H3 Isolated from in-Vitro Reconstituted and Acetylated Nucleosome Core Particle
Source: Mass Spectrom (Tokyo). 2020 Oct 31;9(1):A0090. doi: 10.5702/massspectrometry.A0090 (PMC7674858; doi:10.5702/massspectrometry.A0090)
Supplement: Supplementary Data [file massspectrometry-9-1-A0090-s001.pdf]

Mass spectrometric characterization of histone H3 isolated from in-vitro reconstituted and acetylated nucleosome core particle

Kazumi Saikusa[1, 2, 3]*, Haruna Hidaka[2], Shunsuke Izumi[2,4], and Satoko Akashi[3]

[1] National Metrology Institute of Japan (NMIJ), National Institute of Advanced Industrial Science and Technology (AIST), 1-1-1 Umezono, Tsukuba, Ibaraki 305-8563, Japan

[2] Graduate School of Science, Hiroshima University, 1-3-1 Kagamiyama, Higashi-Hiroshima, Hiroshima 739-8526, Japan

[3] Graduate School of Medical Life Science, Yokohama City University, 1-7-29 Suehiro-cho, Tsurumi-ku, Yokohama, Kanagawa 230-0045, Japan

[4] Graduate School of Integrated Sciences for Life, Hiroshima University, 1-3-1 Kagamiyama, Higashi-Hiroshima, Hiroshima 739-8526, Japan

Supplementary File

Contents

Figures S-1, S-2, S-3

Supplementary Figures

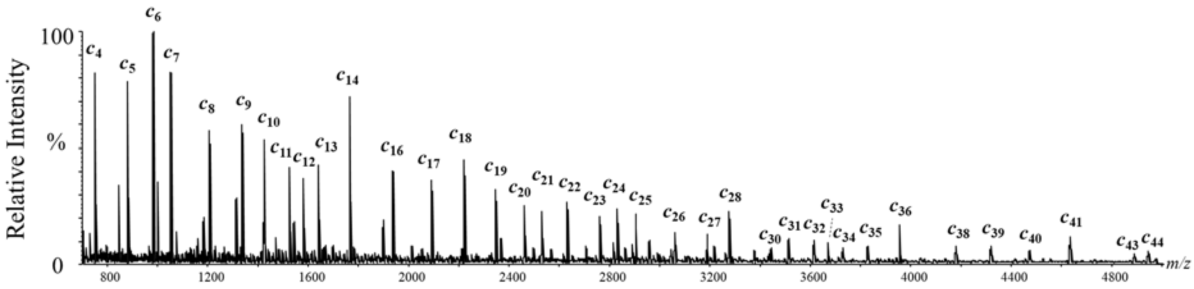

Figure S-1. MALDI-ISD mass spectrum of recombinant H3 in the range of *m/z* 700-5000. The numbers used for annotation of *c* ions start from Ala1 in the original H3 sequence shown in Figure 1.

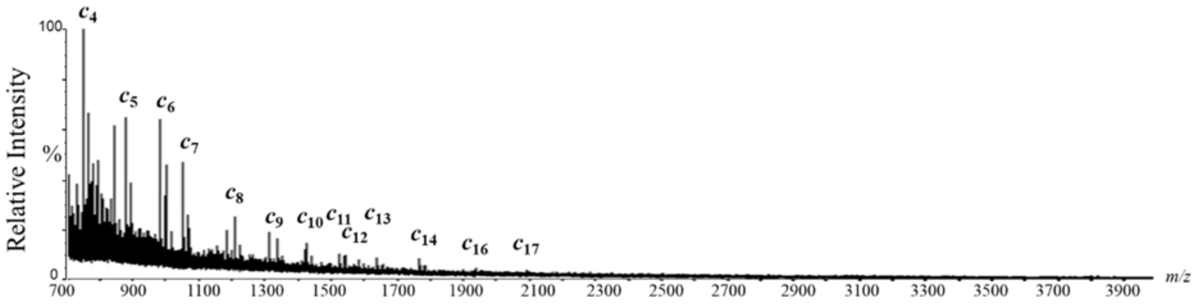

Figure S-2. MALDI-ISD mass spectrum of H3 isolated from reconstituted NCP.
The mass spectrum was obtained by Biflex IV. The numbers used for annotation of *c* ions start from Ala1 in the original H3 sequence shown in Figure 1.

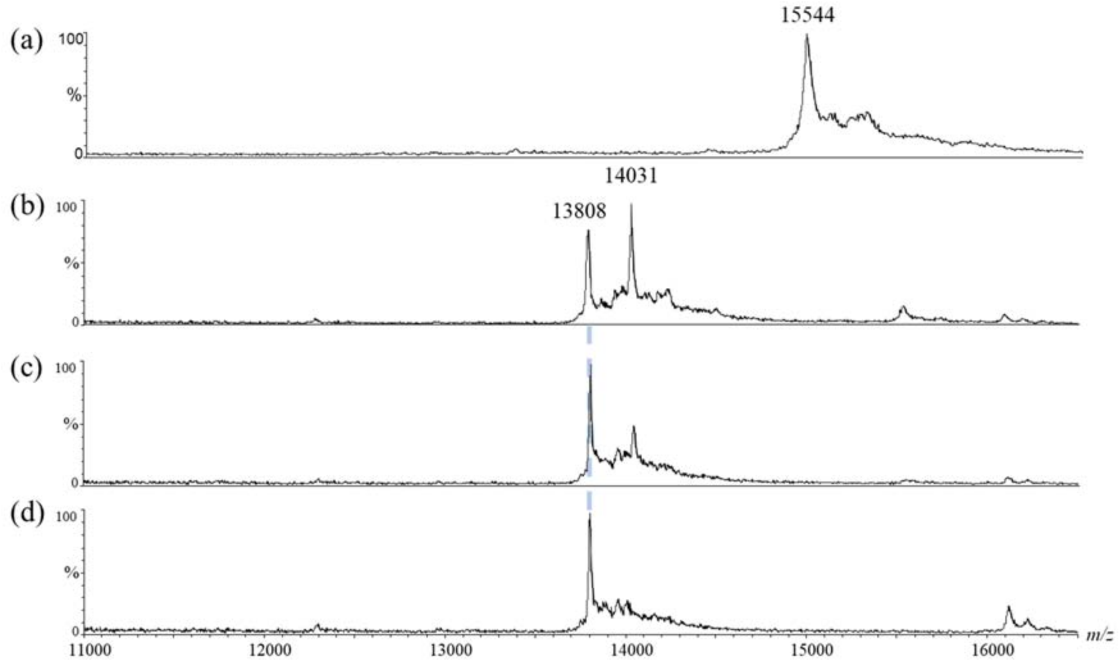

Figure S-3. MALDI mass spectra of aminopeptidase digested H3.

Reaction time:(a) 10 min, (b) 240 min, (c) 360 min, (d) 1 day. The mass spectra were obtained by AXIMA-CFR.
